# Inhibition of prostate cancer cell line (PC-3) by anhydrodihydroartemisinin (ADHA) through caspase-dependent pathway

**DOI:** 10.17179/excli2020-1331

**Published:** 2020-05-11

**Authors:** Faiz Ahmad, Amit Sarder, Rajesh Gour, Shibendra Kumar Lal Karna, Priya Arora, K. P. Ravindranathan Kartha, Yuba Raj Pokharel

**Affiliations:** 1 Faculty of Life Sciences and Biotechnology, South Asian University, New Delhi-110021, India; 2 Department of Medicinal Chemistry, National Institute of Pharmaceutical Education and Research, S.A.S Nagar, Punjab-160062, India

**Keywords:** anhydrodihydroartemisinin, PC-3, apoptosis, caspase 3, caspase 7, c-Jun, Akt, NF-kappaB

## Abstract

Cancer is a generic term for a large group of diseases characterized by the growth of abnormal cells, which is the second leading cause of death globally. To treat cancer, currently, a number of anticancer drugs belonging to various classes chemically are available. The discovery of artemisinin and its derivatives such as artesunate, arteether, and artemether became a milestone in the cure for malaria. Here, we report the anti-cancer property of anhydrodihydroartemisinin (ADHA) - a semisynthetic derivative of artemisinin against prostate cancer cell line PC-3. ADHA was found to be inhibiting growth of PC-3 cells. ADHA was also found to be inhibiting migration of PC-3 cells. At molecular level, ADHA was found to be inhibiting the expression of c-Jun, p-c-Jun, p-Akt and NF-κB and activated caspase 3 and 7. The results show that ADHA like few other artemisinin derivatives hold potential to be used as an anti-cancer agent against prostate cancer cells.

## Introduction

**Figure 1:**
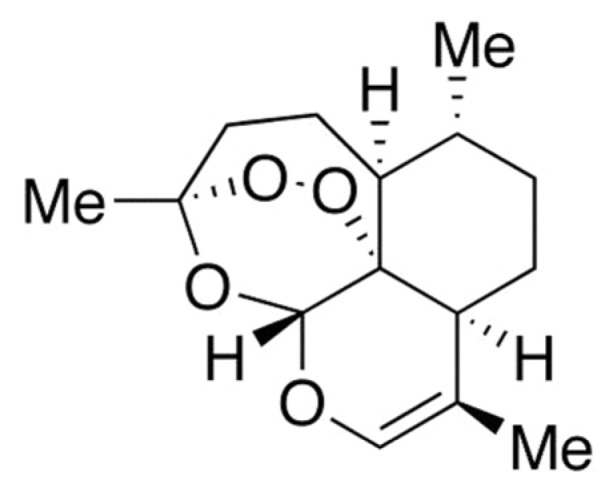
Chemical structure of anhydrodihydroartemisinin (ADHA)

Artemisinin (ART) is basically a natural endoperoxide containing sesquiterpene isolated from one kind of Chinese medicinal plant *Artemisia annua* L. (Qinghao). ART, also known as Qinghaosu, was discovered by Tu Youyou in the early 1970s and the structure of ART was determined in 1979 (Buragohain et al., 2014[3]; Zhu et al., 2014[27]; Weathers et al., 2006[24]). ART is an established antimalarial with potent anticancer activity (Fox et al., 2016[6]). Recently, anticancer activity of ART against cancer cells including prostate cancer cells, breast cancer cells, colon cancer cells, leukemia cancer cells and ovarian cancer cells have been reported. Also, there are evidences that ART is capable of inducing apoptotic cell death (Button et al., 2014[4]). Although the mechanism of action of ART is not clearly understood, the anticancer property of ART is attributed to the bioactivation of endoperoxide bond (Fox et al., 2016[6]; Button et al., 2014[4]). Iron and heme or heme-bound proteins are found to be involved in the bioreductive activation of ART. Cancer cells contain higher amount of intracellular free iron than normal cells because growth and proliferation of cancer cells require high iron metabolism. Thus, cancer cells express increased amount of transferrin receptors (TfR) for uptaking of iron and for regulating the intracellular concentrations of iron. Iron-activated ART is capable of inducing damage by releasing radical oxygen species (ROS) and carbon-centered radicals (Crespo-Ortiz and Wei, 2012[5]; Lai et al., 2013[14]). Despite being a good anti-malarial agent and having potent anti-cancer properties, the therapeutic value of ART is limited by several drawbacks. The drawbacks of ART include its low solubility in both water and oil, its shorter half-life etc. These limitations encouraged the development of new synthetic or semi-synthetic derivatives of ART with better pharmacological properties (Brown, 2010[2]; Li and Zhou, 2010[15]). The most important derivatives of ART include artesunate (ARS), artemether (AM), arteether (AE), dihydroartemisinin (DHA), anhydrodihydroartemisinin (ADHA, Figure 1(Fig. 1)) etc. which exhibit greater efficacy and potency than the parent ART molecule (Sarder and Pokharel, 2016[20]; Meshnick et al.,1996[17]).

Since the discovery of ART, efforts have been made on the chemical modifications of it to improve the pharmaceutical value (Grellepois et al., 2001[7]). Thus, ADHA, a semi-synthetic derivative of ART, has been prepared from the DHA. As an antimalarial agent, improved and favorable properties of ADHA over the parent ART molecule have been reported (Khalifa et al., 1994[12]; Grellepois et al., 2002[8], 2004[9]). But, the anticancer properties of ADHA is yet to be studied. So, the present study was designed to study the effects of ADHA against PC-3 cells.

## Materials and Methods

PC-3 cell line was purchased from the National Center for Cell Science, Pune, India and was cultured in RPMI-1640 (Invitrogen) supplemented with 10,000 units/mL penicillin and 10 mg/mL streptomycin (HIMEDIA) and 10 % heat-inactivated FCS (Invitrogen). The antibodies against c-Jun and P-c-Jun were purchased from Abcam. The anti-caspase 3, anti-caspase 7, anti-NF-κB and anti-p-Akt were purchased from Santa Cruz. Bio-Rad Clarity™ Western ECL substrate was purchased from Bio-Rad Laboratories. ADHA was obtained from National Institute of Pharmaceutical Education and Research, Mohali from Prof. KPR Kartha’s Laboratory.

### Crystal violet assay

The *in vitro* cell viability was evaluated by crystal violet (CV) assay. Briefly, cells were seeded in 96-well plate; 5000 cells per well followed by exposure to ADHA for 72 h in a dose-dependent manner (vehicle control, 0.1 µM, 0.3 µM, 1 µM, 3 µM and 10 µM). This incubation period was followed by removal of media and staining of cells for 30 min with 0.4 % crystal violet. The wells were washed to remove excess dye and then the plate was allowed to dry overnight. Next day, dye was dissolved in methanol and absorbance was measured at 540 nm.

### Colonogenic assay

Colonogenic assay was done with PC-3 cells. For the assay, 500 cells/well were plated in the six-well plate. Once the cells were attached, cells were exposed to ADHA in a dose-dependent manner. After that, the cells were fixed with methanol and stained with 0.4 % crystal violet and colonies were counted using imageJ software.

### Wound healing assay

Approximately 3 X 10^5^ PC-3 cells were seeded in each well of a six-well plate and the cells were allowed to form a monolayer. The monolayer so formed was given a scratch to create a gap. The movement of cell front was photographed at 0 and 12 h. Analysis of the wound area was done by imageJ software.

### Western blotting 

PC-3 cells treated with ADHA were harvested after a 72 h incubation period and lysed in 2X SDS lysis buffer containing 0.5 M Tris-HCl, pH 6.8, glycerol, 10 % (w/v) SDS, protease inhibitor cocktail. The lysates were sonicated and centrifuged at 16000 g for 20 min following which supernatant was collected and subjected to protein estimation by BCA method. 30-40 µg of proteins were separated by SDS gel and transferred onto PVDF membranes. Membranes were blocked with 5 % skim milk followed by incubation with primary antibodies kept at 4 ºC for overnight and with secondary antibody for 1 h. Proteins were detected using chemiluminescence.

### Statistical analysis

The results are expressed as the mean ± standard deviation (SD). A paired t-test was used to determine the significant difference between groups. A p-value < 0.05 was considered as significant.

## Results

### PC-3 cell viability is inhibited by ADHA

**Figure 2:**
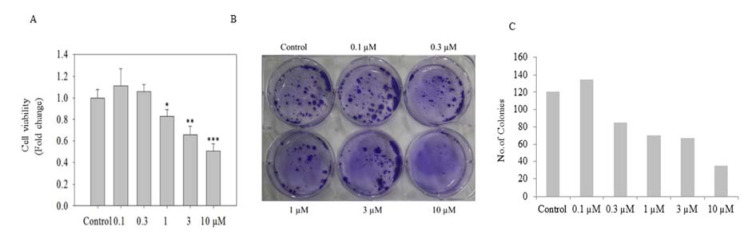
*In vitro* cell viability assay of PC-3 cells. (A) The graph shows cell viability of PC-3 cells seeded in 96-well plate at 5000 cells per well assessed by CV assay after exposure to ADHA for 72 h. (B) Colonogenic assay: PC-3 cells seeded 500/well were assayed for colony forming ability in the presence of ADHA. (C) The graph shows the no. of colonies produced in the colonogenic assay after treatment with ADHA in dose-dependent manner. Results are expressed as fold change ± S. D. * denotes P < 0.05, ** denotes P <0.01 and *** denotes P <0.001.

PC-3 cells treated with ADHA were subjected to crystal violet assay. As shown in Figure 2A(Fig. 2), ADHA significantly reduced cell viability particularly at 1, 3 and 10 µM. Colonogenic assay showed the inhibitory effect of ADHA on the ability of PC-3 cells to grow in colonies as shown in Figure 2B(Fig. 2) which clearly showed the cytostatic property of ADHA against PC-3 cells. We observed a marked reduction in the colony numbers of PC-3 cells with highest concentration; 10 µM of ADHA as shown in Figure 2C(Fig. 2). The colony numbers were evaluated using the ImageJ software.

### ADHA significantly reduced cell migration of PC-3 cells

**Figure 3:**
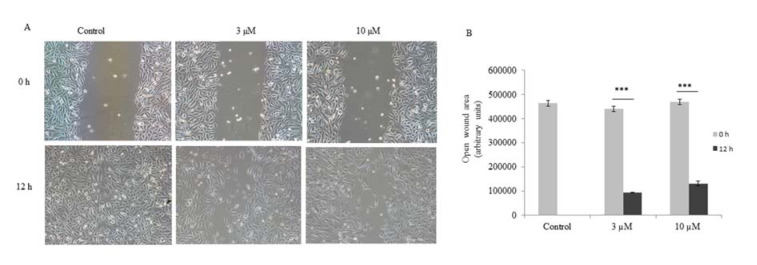
ADHA inhibited migation of PC-3 cells. (A) Representative images of wound healing assay carried on PC-3 cells treated with ADHA. (B) A significant open wound area was observed after 12 h time point in both 3 and 10 µM ADHA treated PC-3 cells. Results are expressed as fold change ± S. D. *** denotes P <0.001.

Since PC-3 cells are metastatic in nature, we checked the anti-migratory property of ADHA. Our result showed that ADHA significantly inhibited the migration of PC-3 cells at both 3 and 10 µM as shown in Figure 3A(Fig. 3). Images of the cell migration were captured at indicated time points and analyzed by imageJ software.

### ADHA inhibits c-Jun, p-c-Jun, NF-κB, p-Akt and activates intrinsic apoptosis 

**Figure 4:**
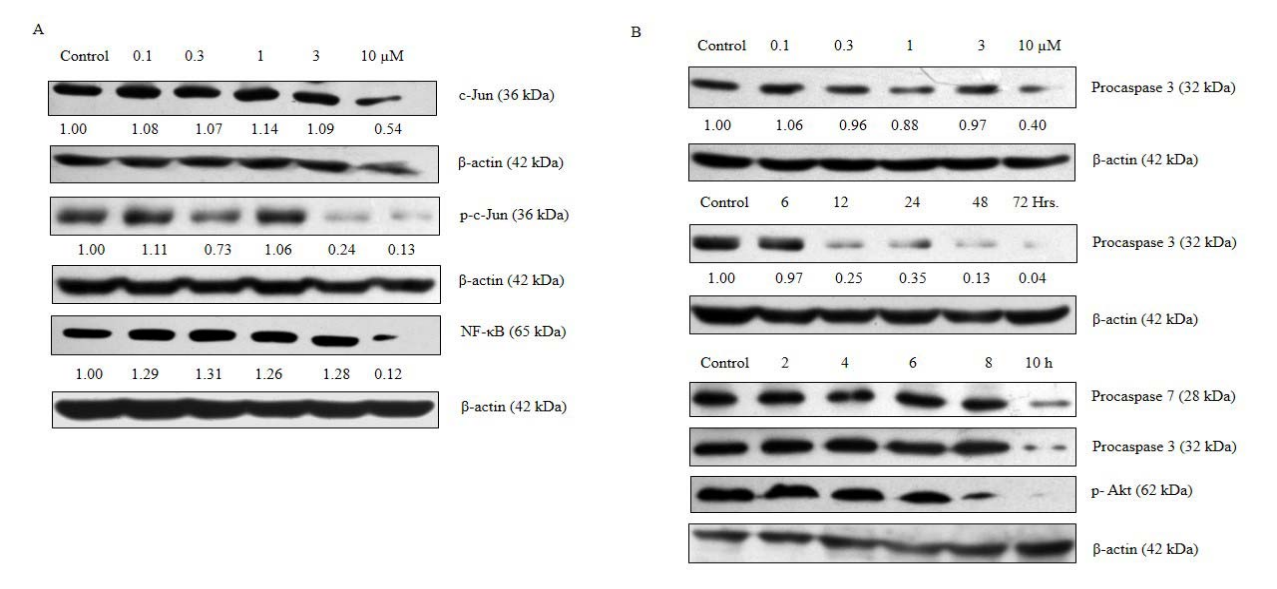
ADHA caused inhibition of c-Jun, p-c-Jun, p-Akt, NF-*κ*B and activation of procaspase 3 and procaspase 7 in PC-3 cells. PC-3 cells were cultured in six-well plate and treated with increasing concentrations of ADHA or treated with 10 µM concentrations of ADHA at different time points. (A) Inhibition of c-Jun, p-c-Jun and NF-*κ*B treated with ADHA. (B) ADHA caused decreased expression of procaspase 3 and 7 and p-Akt in dose- and time-dependent manner wherever specified.

We studied the effect of ADHA on the expression of c-Jun, p-c-Jun and NF-*κ*B and checked the expression of markers associated with intrinsic mode of apoptosis. We found that ADHA has a suppressive effect on the expression of the c-Jun, p-c-Jun and NF-*κ*B particularly at 10 µM as shown in Figure 4A(Fig. 4). We also observed the impact of ADHA on the activation of the procaspase 3 in a dose- and time-dependent manner (6, 12, 24, 48, and 72 h at 10 µM ) and found that activation of procaspase 3 came into effect from 12 h time point which gradually increased up to 72 h as shown in Figure 4B(Fig. 4). Next, we checked the activation of caspase 3 as well as of caspase 7 in a time-dependent manner (2, 4, 6, 8, 10 h at 10 µM) and found that ADHA caused activation of procaspase 3 and 7 at 10 µM as shown in Figure 4B(Fig. 4). Following the same set of condition as done for caspase 3 and 7, we also checked the phosphorylation status of Akt as shown in Figure 4B(Fig. 4). ADHA caused inhibition of phosphorylated Akt at 10 h time point as shown in Figure 4B(Fig. 4) which reflects the potential of ADHA as an anti-proliferative agent.

## Discussion

Artemisinin derivatives are being studied worldwide for their efficacy against a number of diseases, primarily for malaria. We here, report the anti-cancer effect of an artemisinin derivative; ADHA. Although the anti-leishmanial and anti-malarial activity have been reported for the ADHA (Slade et al., 2009[21]), its anti-cancer property to our best knowledge has not been explored. In the present study, we found that ADHA caused a dose-dependent inhibition of PC-3 cells and reduced the growth of single cell and thus inhibited their ability to form a larger colony particularly at 3 and 10 µM w.r.t control as assayed by CV and colonogenic assay respectively which reflect cytotoxic property of ADHA. Previous studies have shown the anti-migratory effect of artemisinin and its various other derivatives (Ju et al., 2018[11]; Tong et al., 2016[23]). To determine the anti-metastatic effect of ADHA on metastatic PC-3 cells we conducted wound healing assay and found that ADHA significantly halted cell migration at 3 and 10 µM w.r.t control. 

In view of identifying the molecular markers, we checked the expression of c-Jun and phospho-c-Jun, the latter is a positive regulator of cell cycle. It can promote cell growth and can also transduce a mitogenic response. It also plays a role in cell proliferation, migration and invasion of cancer (Zhang et al., 2007[26]; Mialon et al., 2005[18]; Lukey et al., 2016[16]). ADHA was found to be inhibiting both phospho and total c-jun.

Next, we checked a time-dependent expression of phospho-Akt which is a proven survival factor and can exert anti-apoptotic effects by preventing the release of cytochrome c from mitochondria. It phosphorylates and inactivates the proapoptotic factor BAD and procaspase 9. And, also it phosphorylates and inactivates the transcription factor FOXO which regulates the expression of genes important for apoptosis (Altomare and Testa, 2005[1]; Kumar et al., 2013[13]). Again, it can also activate IκB kinase, a positive regulator of NF-κB, which leads to the transcription of anti-apoptotic genes (Testa and Bellacosa, 2001[22]). ADHA showed an inhibitory effect on the expression of phospho-Akt and expression was found to be downregulated after 10 h of ADHA treatment. 

Again, NF-κB plays a crucial role in cancer initiation and progression. It regulates a variety of cellular processes including cell proliferation. It plays a role in cancer progression by regulating the epithelial to mesenchymal transition (Hoesel and Schmid, 2013[10]; Xia et al., 2014[25]). Constitutively activated NF-κB is associated with prevention of apoptosis and increasing angiogenic and metastatic potential (Park and Hong, 2016[19]). 10 µM of ADHA was found to be inhibiting NF-κB which shows the potential of this derivative to be used as a cytostatic or cytotoxic agent against prostate cancer.

Finally, we can conclude that ADHA has cytotoxic effects on PC-3 cells and causes cell death via apoptosis of PC-3 cells via caspase-dependent intrinsic signaling cascade.

## Notes

Faiz Ahmad and Amit Sarder contributed equally to this manuscript.
